# Awareness and Attitude of the General Population Towards Inherited Hemoglobinopathies in the Premarital Screening Program in the Northern Region of Saudi Arabia

**DOI:** 10.3390/hematolrep17010009

**Published:** 2025-02-05

**Authors:** Mariah N. Hafiz, Nida Suhail, Zakariya M. S. Mohammed, Husham O. Elzein, Hibah A. Almasmoum, Awad E. Abass, Mohammed M. Jawad, Saoussen Trabelsi

**Affiliations:** 1 Department of Medical Laboratory Technology, Faculty of Applied Medical Sciences, Northern Border University, Arar 91431, Saudi Arabia; mariah.hafiz@nbu.edu.sa (M.N.H.); husham.osman@nbu.edu.sa (H.O.E.); awad.mahmoud@nbu.edu.sa (A.E.A.); mohammed.jawad@nbu.edu.sa (M.M.J.); 2 Center for Scientific Research and Entrepreneurship, Northern Border University, Arar 91431, Saudi Arabia; zakariya.mohammed@nbu.edu.sa; 3 Department of Mathematics, College of Science, Northern Border University, Arar 91431, Saudi Arabia; 4 Department of Clinical Laboratory Sciences, Faculty of Applied Medical Sciences, Umm Al-Qura University, Makkah 7607, Saudi Arabia; hamasmoum@uqu.edu.sa; 5 Department of Community Health, Faculty of Applied Medical Sciences, Northern Border University, Arar 91431, Saudi Arabia; sawsan.trablsi@nbu.edu.sa; 6 Centre for Health Research, Northern Border University, Arar 91431, Saudi Arabia

**Keywords:** awareness, hemoglobinopathies, Northern Border Region, premarital screening

## Abstract

**Background:** Premarital screening (PMS) is a nationwide program that helps high-risk individuals make decisions to avoid genetic and sexually transmitted diseases from spreading to their spouse or future offspring. This study examined the knowledge and attitudes towards inherited hemoglobinopathies in PMS among the people of Northern Border Region in Saudi Arabia and their relationship to various sociodemographic factors. **Methods:** A cross-sectional study was undertaken in the Northern region of Saudi Arabia from January to March 2024. Data were gathered via questionnaire from 478 Saudi participants aged 18 years and older. The chi-square test was employed to determine the association between categorical variables. **Results:** All participants in the study were familiar with the PMS program. A significant portion of participants, 79.3%, acknowledged that consanguinity can increase the risk of hereditary blood disorders, while 69.9% believed that if both parents are carriers of the same genetic blood disease, their child may inherit it. Higher education, female gender, and age group (30–40) were found to be the main predictors of knowledge regarding PMS. Most of the participants (98.5%) had a positive attitude regarding the necessity of PMS as a prerequisite for marriage completion. About 82.8% indicated they would not continue with the marriage if the PMS results were incompatible. **Conclusions:** The study indicates a growing awareness and positive attitude towards premarital screening among the general population, with an increasing number of individuals opting for it. The findings suggest that PMS programs contribute to informed decision making, as evidenced by the rise in participants choosing to forgo marriage due to partner incompatibility. The study recommends the enhancement of health education campaigns by considering demographic factors such as age, education, and marital status. Additionally, it advocates for expanding the scope of PMS to include a wider range of health and genetic disorders to improve its overall efficacy.

## 1. Introduction

Hemoglobinopathies, including thalassemia and sickle cell disease (SCD), are among the most common inherited disorders globally, significantly impacting mortality and morbidity [1,2]. According to the World Health Organization (WHO), at least 5% of the world’s population is a carrier for genetic disorders, particularly hemoglobinopathies (2.9% for thalassemia and 2.3% for sickle cell anemia) [3]. Sickle cell disease is characterized by mutations in the gene that encode the beta subunit of hemoglobin. It is an autosomal recessive disorder requiring both parents to transmit a defective gene for the child to manifest the disease. It results in sickle-shaped red blood cells that block blood flow, leading to painful complications. The pathophysiology of thalassemia entails a disturbance in the equilibrium of globin chain synthesis, resulting in an imbalance between alpha- and beta-globin chains and associated anomalies in red blood cell production and functionality. Individuals with β-thalassemia exhibit impaired erythropoiesis, necessitating lifelong blood transfusions [4].

Research indicates that prevalence of autosomal recessive disorders is higher in populations where consanguinity is common [5]. Consanguineous marriages, where couples are genetically related, are more common in Saudi Arabia compared to Western European and Asian nations, with an estimated prevalence of 56%, especially in rural areas [6]. Such marriages increase the risk of autosomal recessive disorders by amplifying the expression of deleterious genes, reducing genetic diversity within families and communities [7]. This reduction in diversity may lead to broader health issues, such as weakened immunity, greater susceptibility to infectious diseases, and a higher incidence of multifactorial diseases like heart disease and diabetes, which are influenced by both genetic and environmental factors [8].

SCD and thalassemia are major public health issues in the Kingdom, imposing huge health and economic burdens on both society and the affected individuals. Saudi Arabia has one of the highest prevalence rates of β-thalassemia and SCD among Middle Eastern countries (0.05% and 4.50%, respectively) [9]. According to the General Statistical Organization, the estimated prevalence of beta-thalassemia and SCD in Saudi Arabia is 1–5% and 17%, respectively [10,11]. To reduce the burden of these hereditary disorders, the premarital screening (PMS) program was initiated in Saudi Arabia in 2001; however, it became mandatory only in 2004 [12,13]. It involves screening couples intending to get married for hemoglobinopathies such as sickle cell anemia, thalassemia, and viral infectious diseases such as HIV and hepatitis B and C [14,15]. Currently, it is being conducted in 130 Ministry of Health hospital centers throughout the Kingdom [16]. Screening seeks to assess the probability of disease transmission to the spouse or offspring and to offer partners alternatives for planning a healthy family [17,18]. The PMS program has effectively contributed to alleviating the challenges associated with hemoglobinopathies by decreasing the incidence of high-risk marriages [9,13]. The effectiveness of the PMS program is demonstrated by a decrease in the percentage of high-risk couples who chose to marry, from approximately 90% at the end of 2005 to 73% by the end of 2009 [13]. Furthermore, an examination of the decrease in the proportion of identified risky couples from 2.14% at the end of 2005 to 1.13% at the end of 2009 suggests that increased public health awareness regarding hereditary and infectious diseases has contributed to a decline in marriage proposals among couples known to carry or be affected by such conditions [13].

Health education is the best way to improve the situation of any community with a higher incidence of certain practices such as consanguinity to prevent the occurrence of many inherited diseases, including the hemoglobinopathies [10]. For effective education, the knowledge degree and changing attitude of the general population towards PMS need to be assessed in advance. This can be performed using community-based surveys [10,19].

Several studies in Saudi Arabia have evaluated people’s knowledge and attitudes towards premarital screening [20,21,22]. However, most of these studies were focused on regions with a higher prevalence of hemoglobinopathies [14]. To the best of our knowledge, no research has been conducted to assess people’s awareness and attitudes concerning PMS in Saudi Arabia’s Northern Border Region, a sparsely populated and emerging area. Consequently, it is essential to determine whether the residents in this region lack awareness and require education, or if their understanding of the significance of PMS before marriage is adequate. This study was conducted to assess the knowledge and attitudes of Saudi citizens towards PMS in the Northern Region of Saudi Arabia and to associate this with various sociodemographic factors (such as gender, age, education level, and marital status). Identifying the associated sociodemographic factors could open the door for stakeholders seeking to target education about this program within Saudi Arabia.

## 2. Materials and Methods

### 2.1. Data Collection

A descriptive cross-sectional study was undertaken on the general Saudi population aged 18 years and above in the Northern region of Saudi Arabia between January 2024 to March 2024. Data were collected through a questionnaire which was created using Google Forms. The questionnaire was circulated among the general population of the region electronically via text messages and social media platforms such as WhatsApp and Twitter. All participant information was kept confidential. Names and identification details were not sought. Participation was entirely voluntary, and participants were allowed to leave the survey at any point. Completing the survey was regarded as informed consent to participate.

The minimum required sample size (385) was calculated using an online calculator (Qualtrics) considering a margin of error of 5% and a confidence level of 95%. However, we aimed to gather as many responses as possible from the general population of the region. The study enrolled 478 participants comprising both males and females.

The questions were initially written in English and then translated into Arabic, which is the region’s native language. Experts verified and checked the survey questions for content validity. Participants who failed to finish the survey were excluded. The questionnaire consisted of three parts: sociodemographic data, knowledge, and attitude questions about PMS.

Part 1: Sociodemographic characteristics consisted of 5 items, which included age, gender, educational level, marital status, and place of residence.

Part 2: The knowledge domain included 6 items to assess the participant’s knowledge regarding the genetic diseases and PMS program (including transmission of the disease, risk factors, importance of PMS program, and the diseases covered by the program). A three-point Likert scale (agree, disagree, and undecided) was used to assess this domain.

Part 3: This part comprised 5 items and assessed the participants’ attitude toward PMS. The participants were classified as having positive or negative attitude based on their answers (correct answer: positive attitude, incorrect answer: negative attitude).

### 2.2. Inclusion and Exclusion Criteria

Saudi males and females above 18 years of age and belonging to the Northern Border Region (which includes small cities such as Rafha, Turayf, and Al Uwayqilah, in addition to the capital city of Arar) were included in the study. Participants under 18 years of age, non-Saudis, and those who were not willing to participate in the study were excluded.

### 2.3. Statistical Analysis

Data entry and analysis was performed using Statistical Package for Social Sciences (SPSS) version 26. Descriptive statistics were performed using frequencies and percentages for categorical variables. The chi-square test was used to assess the relationship between sociodemographic factors and knowledge and attitudes of the people towards PMS. A *p* value of <0.05 was considered statistically significant.

## 3. Results

Only the items related to knowledge and attitude that demonstrated a significant association with sociodemographic factors are included in the tables and figures of the main article. Kindly consult the Supplementary Tables for comprehensive data.

### 3.1. Sociodemographic Profile

The sociodemographic characteristics of the participants are listed in Table 1. The study enrolled 478 participants, comprising 405 female participants (84.7%), compared to only 73 male participants (15.3%). The majority of the respondents were in the age group 18–29 (59.2%) and belonged to Arar (79.9%). Based on the marital status, 217 (45.4%) were previously married, whereas 261 (54.6%) had never been married. The majority of participants (97.3%) had a university degree or higher. Approximately 21.1% of individuals had consanguineous marriages, out of which 68 (67.3%) were married to their first cousins (first-degree relative) and 33 (32.7%) were married to other relatives (second-degree relative). Family history of the genetic disease was reported by 49 (10.3%) participants and personal history of the hereditary disease was reported by 26 (5.4%) participants. Family/cultural pressure was identified as the primary reason by most of the participants (38.7%) for proceeding with the marriage despite incompatible PMS results and a significant risk of offspring with a genetic blood disorder. Nearly all respondents (98.1%) were aware of PMS and 79.3% indicated a necessity for additional information regarding the PMS program to respond to enquiries pertaining to premarital screening with clarity (Supplementary Table S1).

### 3.2. Knowledge

**Figure 1:**
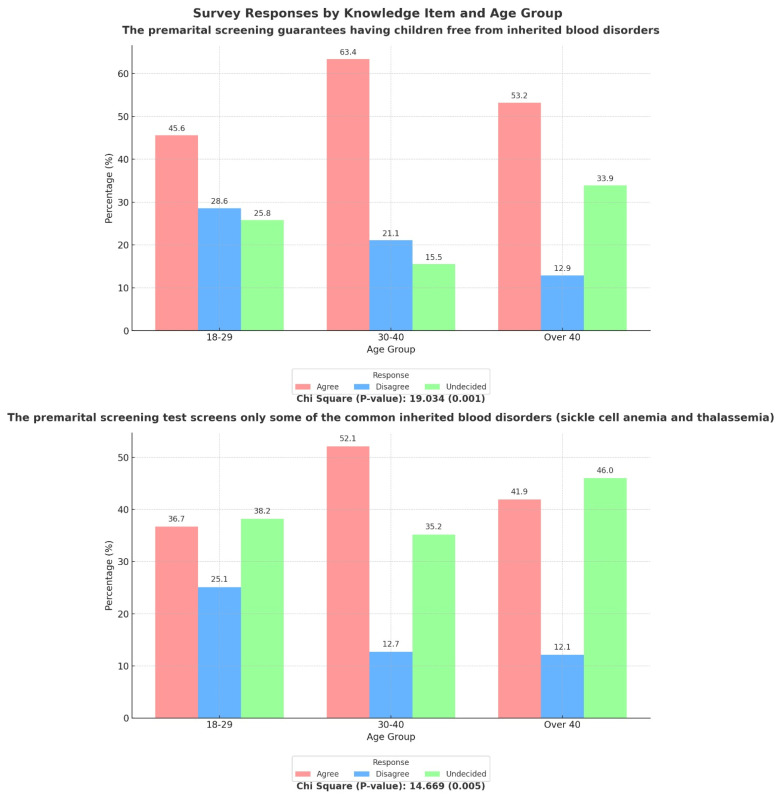
Association between participants’ knowledge of PMS test and age group.

**Figure 2:**
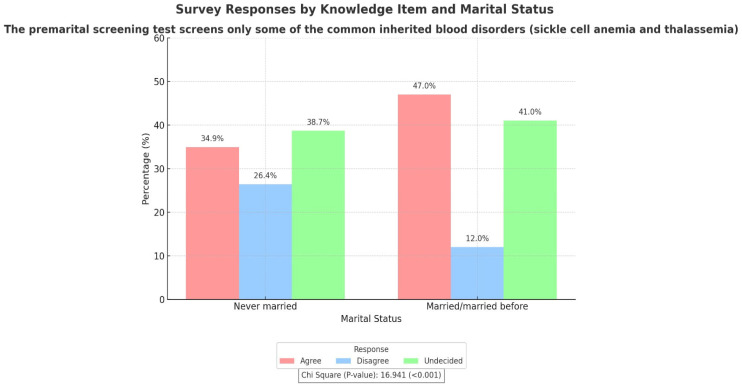
Association between participants’ knowledge of PMS test and marital status.

The knowledge of participants regarding PMS was assessed by several questions listed in Supplementary Table S2 and its association with various sociodemographic determinants is illustrated in Table 2 and Table 3 and Figure 1 and Figure 2. Nearly half of the study population (50.2%) agreed that premarital screening ensures that children are free of inherited blood disorders. The majority of the participants (40.4%) agreed that PMS test screens only some of the common inherited blood disorders (sickle cell anemia and thalassemia), but almost equal number (39.7%) of respondents were not aware about it. This agreement was significantly associated with the age group (*p* = 0.005) of participants and their marital status (*p* < 0.001), where people in the age range of 30–40 years and who were married/married before had greater knowledge (Figure 1 and Figure 2). The study population was also aware (79.3%) of the fact that consanguinity can raise the risk of inherited blood diseases in children. Females (*p* = 0.002) and people with university degrees (*p* = 0.010) showed significant awareness as compared to the others (Table 2 and Table 3). When asked about their knowledge of disease transmission, more than half of the respondents (69.9%) were convinced that if both parents are carriers for the same genetic blood disease, there will be a risk of having a child affected with this disorder. Higher education level (*p* = 0.007) and female gender (*p* = 0.009) were found to be significantly associated with the knowledge about disease transmission (Table 2 and Table 3). The majority of the participants (56.5%) were also aware that, if the couples were both carriers of a specific genetic blood disorder, there is a procedure called pre-implantation genetic testing that can be considered instead of separation. This awareness was significantly correlated with gender (*p* = 0.018), where females were more aware than males (Table 2).

### 3.3. Attitude

The questions included in Supplementary Table S3 were used to gauge participants’ attitudes towards PMS and their association with sociodemographic factors is shown in Table 4, Table 5 and Table 6. In general, most participants (98.5%) had a positive attitude towards PMS and believed that premarital screening should be mandatory before the completion of a marriage. The majority of the respondents (76.2%) agreed that, if the couples were at a high risk of having children with a genetic blood disorder, their marriage should be prohibited; only 23.8% of people disagreed with this statement. People above 40 years of age (*p* = 0.023) and who were married/married before (*p* = 0.036) displayed higher positive attitudes showing significant association with this statement (Table 5 and Table 6). Participants were also asked if they would continue with the marriage if the person they wanted to marry was a carrier of one of the genetic blood diseases while they themselves were healthy, or if both of them were carriers; the majority of them (77% and 82.8% respectively) refused, while only a small percentage (23% and 17.2% respectively) agreed to complete the marriage in such cases (Supplementary Table S3). These attitudes showed significant association with gender (Attitude item 1—*p* = 0.030 and Attitude item 2—*p* = 0.001), age (Attitude item 2—*p* = 0.015 and Attitude item 3—*p* < 0.001) and marital status (Attitude item 2—0.006) with females, people over 40 years, and those who were married/married before exhibiting higher degrees of positive attitude (Table 4, Table 5 and Table 6). Furthermore, about 93.7% of the participants in the survey supported the notion of incorporating tests that screen for all potential hereditary blood disorders to expand the scope of the premarital screening program. This attitude was significantly associated (*p* = 0.012) with the marital status of participants, where people who were married/married before had a higher rate of positive attitude (Table 6).

## 4. Discussion

This study assessed the knowledge levels and attitudes associated with PMS among the people of Northern Border Region in Saudi Arabia and examined the associated sociodemographic characteristics (such as gender, age, education level, and marital status). Four hundred and seventy-eight individuals participated in the study, with females making up approximately 84% of the participants, likely due to their greater concern for future generations, as well as their active and cooperative nature. The current study found consanguinity in 21.1% of subjects, with 67.3% being first-degree relatives. Consanguinity is a very common practice in the Arab countries. The estimated prevalence of consanguinity in Saudi Arabia is 56%, with a markedly greater incidence in rural areas than in metropolitan regions [6]. The percentage in our study is substantially lower than that reported by earlier studies [6,23]. This shift in trend suggests that increased public health awareness regarding hereditary and infectious diseases through health education initiatives and the PMS program might have contributed to a decline in marriage proposals among couples known to carry or be affected by such conditions.

### 4.1. Knowledge

Almost all participants (98.1%) in the study were familiar with the PMS program. The majority of respondents (40.4%) agreed that the PMS test screens only some common inherited blood disorders, such as sickle cell anaemia and thalassaemia, whereas a nearly equivalent proportion (39.7%) were uninformed about the diseases screened by the PMS program. This disparity in knowledge can be ascribed to the differing levels of exposure to various information sources among these groups. It underscores the necessity to enhance the public awareness about PMS and screened genetic and infectious diseases associated to it. Our findings align with numerous prior research undertaken in Saudi Arabia, within particular population groups. A study revealed that unmarried female university students have insufficient information regarding PMS, with fewer than one-third of participants aware of the diseases assessed and only 6.6% demonstrating satisfactory knowledge of PMS prior to a health education program [19]. A further study evaluated the Al-Madinah community’s awareness of the PMS program and discovered that fifty percent were uninformed about the disorders being screened [24]. The study found that participants had good knowledge of hereditary risks associated with blood disorders and their potential transmission. About 79.3% recognized that consanguinity increases the risk of hereditary blood disorders in offspring, and 69.9% understood that both parents being carriers of the same genetic disorder could result in an affected child. These results are consistent with a Jeddah study [25], where 84% of university students acknowledged consanguinity as a risk factor for genetic disorders. Additionally, 56.5% of participants were aware of pre-implantation genetic testing as an alternative to separation for carriers of specific genetic blood disorders.

The main predictors of knowledge regarding PMS among the general population were higher education, female gender, and the 30–40 age group. Higher education likely reflects exposure to information on PMS, genetic, and infectious diseases, highlighting the need to introduce such topics in secondary schools. This aligns with previous research showing that individuals with university degrees tend to have better knowledge [26]. Females demonstrated significantly higher PMS knowledge than males, possibly due to their greater concern for chronic illnesses affecting both mothers and children. Additionally, the predominance of female respondents may have introduced bias in the study. These findings are consistent with Alhowiti et al. [10], who also found higher PMS knowledge among female students. The 30–40 age group exhibited the greatest knowledge, likely due to completed education and personal experience with PMS, particularly among married individuals. Recent health education initiatives aimed at enhancing awareness of hemoglobinopathies and PMS among secondary school and college students have had positive outcomes [27,28]. Additionally, individuals who were married or previously married showed greater awareness of the diseases screened by PMS, likely due to their prior experience with mandatory premarital screening.

Overall, participants demonstrated good knowledge of PMS, including awareness of hereditary blood disorder risks, consanguinity’s impact on offspring, and pre-implantation genetic testing as an alternative to separation. However, a significant proportion were unaware of the specific diseases screened by PMS, highlighting the need to increase public awareness of PMS and the genetic and infectious diseases it addresses. The study identified higher education, age (30–40), and female gender as key predictors of knowledge, suggesting that future health education campaigns should target these factors. Additionally, an important limitation of the present study is the disproportionate representation of female respondents, which may introduce gender bias and affect the generalizability of the findings. The underrepresentation of male participants restricts the ability to capture the perspectives and awareness of the broader population. To enhance the external validity of the results, future research should strive for a more balanced representation of both genders, ensuring more comprehensive and generalizable conclusions.

### 4.2. Attitude

In this study, the majority of participants (98.5%) held a positive attitude towards PMS as a necessary requirement for marriage. This favorable attitude may support medical professionals in advising couples with positive screening results. Consistent with these findings, Al-Sulaiman et al. [29] reported a predominantly positive attitude towards PMS among the Saudi population, with most respondents supporting its implementation on all couples across the Kingdom. Similarly, a study of female Saudi students found that most participants viewed PMS positively, recognizing the severity of diseases prevented and the benefits it provides [30].

About 82.8% of participants in our study indicated that they would not proceed with marriage if PMS results were incompatible and there was a significant risk of offspring inheriting a genetic blood disorder. However, 17.2% expressed willingness to continue despite these risks. While these findings are encouraging, the minority who would proceed suggest that cultural barriers still exist. This underscores the need for legislation prohibiting incompatible marriages. A study conducted among university students in Taif found that 82.9% would reconsider marriage in the case of incompatibility [31]. In Riyadh, over 60% of participants in a population-based study agreed that marriage plans should be canceled in the presence of a significant hereditary disease risk [29].

The present study reveals that individuals who were married or previously married (*p* = 0.036) and those over 40 years of age (*p* = 0.023) demonstrated significant positive attitude, perhaps due to their prior participation in premarital screening for couples. Additionally, 93.7% of participants supported the inclusion of tests and screening for all potential hereditary blood disorders, suggesting strong support for expanding the premarital screening program. These findings are promising and indicate a positive response to the introduction of new genetic testing within the healthcare system. The results are consistent with other studies showing that Saudi society generally holds a favorable attitude toward genetic testing, including prenatal and pre-implantation genetic diagnosis [32,33,34].

Overall, participants exhibited a positive attitude towards PMS, which could assist medical professionals in counseling couples with positive screening results. While the majority believed that marriages should be canceled in the case of incompatible PMS results, a small proportion still expressed a willingness to proceed, indicating the presence of cultural barriers that need to be addressed. The implementation of a law prohibiting incompatible marriages is warranted.

The study has several limitations. Firstly, the majority of participants were university students from Arar, the capital city, which may have provided them with more opportunities to engage in health education programs. Additionally, the sample had a predominance of female respondents, introducing potential bias and limiting the generalizability of the findings. The underrepresentation of male participants restricts a comprehensive understanding of the broader population’s perspectives. Future research should aim for more balanced gender representation to improve external validity. Furthermore, due to the cross-sectional design, the study can demonstrate associations but not establish causal relationships between the factors and outcomes.

## 5. Conclusions and Recommendations

The results of this study indicate that awareness and attitudes toward PMS are evolving, with an increasing number of individuals expressing interest in participating in the program. The study highlights a generally positive attitude towards PMS and a notable trend of people opting to forgo marriage due to incompatibility with prospective partners. Several recommendations emerged from the study; given that individuals are aware of the program and generally possess a positive attitude towards PMS, prospective health education campaigns and interventions could be improved by taking into account participants’ age, educational attainment, and marital status. Since the study’s underrepresentation of male participants limits the ability to fully capture the perspectives and awareness of the broader population, future research should aim for a more balanced gender representation to improve the external validity of the findings. Additionally, culturally tailored community health education programs focused on PMS should be developed, particularly emphasizing the risks associated with consanguineous marriages. Lastly, expanding PMS to cover a wider range of health and genetic disorders could further enhance its effectiveness and utility.

## Figures and Tables

**Table 1:** Participants’ sociodemographic characteristics.

Variable	Variable Levels	Count	Percentage
Gender	Male	73	15.3%
	Female	405	84.7%
Age (years)	18–29	283	59.2%
	30–40	71	14.9%
	Over 40	124	25.9%
City	Arar	382	79.9%
	Rafha	29	6.1%
	Turaif	53	11.1%
	Owaiqila	6	1.3%
	Villages in the Northern Border Province	8	1.7%
Education Level	Less than university	13	2.7%
	University or higher	465	97.3%
Marital status	Never married	261	54.6%
	Married/ married before	217	45.4%

**Table 2:** Association between participants’ knowledge of PMS test and gender.

S.No	Knowledge Items	Response	Gender			Chi Square (*p*-Value)
			Male	Female	Total	
			%	%	%	
1	The premarital screening guarantees having children free from inherited blood disorders	Agree	34.2	53.1	50.2	9.555 (0.008)
		Disagree	27.4	22.7	23.4	
		Undecided	38.4	24.2	26.4	
		Total	100.0	100.0	100.0	
2	Marriage to a relative (consanguinity) can lead to an increased risk of inherited blood disease in children	Agree	64.4	82.0	79.3	12.506 (0.002)
		Disagree	8.2	3.0	3.8	
		Undecided	27.4	15.1	16.9	
		Total	100.0	100.0	100.0	
3	When both of the parents are carriers for the same genetic blood disease, there will be a risk for having an affected child with this disorder	Agree	54.8	72.6	69.9	9.424 (0.009)
		Disagree	9.6	5.2	5.9	
		Undecided	35.6	22.2	24.3	
		Total	100.0	100.0	100.0	
4	Marriage could be compatible even if one of the parents is a carrier of the genetic disease	Agree	42.5	27.9	30.1	9.708 (0.008)
		Disagree	20.5	37.8	35.1	
		Undecided	37.0	34.3	34.7	
		Total	100.0	100.0	100.0	
5	Do you know that there is a procedure called preimplantation genetic diagnosis that is considered when both spouses are carriers of a genetic disease?	Yes	43.8	58.8	56.5	5.609 (0.018)
		No	56.2	41.2	43.5	
		Total	100.0	100.0	100.0	

**Table 3:** Association between participants’ knowledge of PMS test and education level.

S.No	Knowledge Items	Response	Educational Level			Chi Square (*p*-Value)
			Less Than University	University or Higher	Total	
			%	%	%	
1	Marriage to a relative (consanguinity) can lead to an increased risk of inherited blood disease in children	Agree	46.2	80.2	79.3	9.125 (0.010)
		Disagree	7.7	3.7	3.8	
		Undecided	46.2	16.1	16.9	
		Total	100.0	100.0	100.0	
2	When both of the parents are carriers for the same genetic blood disease, there will be a risk of having a child affected with this disorder	Agree	30.8	71.0	69.9	9.813 (0.007)
		Disagree	15.4	5.6	5.9	
		Undecided	53.8	23.4	24.3	
		Total	100.0	100.0	100.0	

**Table 4:** Association between participants’ attitude regarding PMS test and gender.

S.No	Attitude Items	Response	Gender			Chi Square (*p*-Value)
			Male	Female	Total	
			%	%	%	
1	If you are in a situation where the person you want to marry is a carrier of one of the genetic blood diseases, such as thalassemia or sickle cell anemia, while you are healthy, you will proceed to complete the marriage	Agree	32.9	21.2	23.0	4.732 (0.030)
		Disagree	67.1	78.8	77.0	
		Total	100.0	100.0	100.0	
2	If you were in a situation where both of you are carriers of one of the genetic blood diseases, you will proceed to complete the marriage	Agree	30.1	14.8	17.2	10.217 (0.001)
		Disagree	69.9	85.2	82.8	
		Total	100.0	100.0	100.0	

**Table 5:** Association between participants’ attitude regarding PMS test and age group.

S.No	Attitude Items	Response	Age				Chi Square *p*-Value)	
			18–29	30–40	Over 40	Total		
			%	%	%	%		
1	If the couples were at a high risk of having children with genetic blood disorder, their marriage should be prevented	Agree	71.	81.7	83.1	76.2		7.507 (0.023)
		Disagree	28.3	18.3	16.9	23.8		
		Total	100.0	100.0	100.0	100.0		
2	If you are in a situation where the person you want to marry is a carrier of one of the genetic blood diseases, such as thalassemia or sickle cell anemia while you are healthy, you will proceed to complete the marriage	Agree	25.8	28.2	13.7	23.0		8.359 (0.015)
		Disagree	74.2	71.8	86.3	77.0		
		Total	100.0	100.0	100.0	100.0		
3	If you were in a situation where both of you are carriers of one of the genetic blood diseases, you will proceed to complete the marriage	Agree	22.6	12.7	7.3	17.2		15.484 (<0.001)
		Disagree	77.4	87.3	92.7	82.8		
		Total	100.0	100.0	100.0	100.0		

**Table 6:** Association between participants’ attitude regarding PMS test and marital status.

S.No	Attitude Items	Response	Marital Status			Chi Square (*p*-Value)
			Never Married	Married/Married Before	Total	
			%	%	%	
1	If the couples were at a high risk of having children with genetic blood disorder, their marriage should be prevented	Agree	72.4	80.6	76.2	4.420 (0.036)
		Disagree	27.6	19.4	23.8	
		Total	100.0	100.0	100.0	
2	If you were in a situation where both of you are carriers of one of the genetic blood diseases, you will proceed to complete the marriage	Agree	21.5	12.0	17.2	7.484 (0.006)
		Disagree	78.5	88.0	82.8	
		Total	100.0	100.0	100.0	
3	You wish to add a test/s that screen/s for all the possible inherited blood disorders so that the premarital screening program becomes wider	Agree	91.2	96.8	93.7	6.286 (0.012)
		Disagree	8.8	3.2	6.3	
		Total	100.0	100.0	100.0	

## Data Availability

The original contributions presented in this study are included in the article/Supplementary Material. Further inquiries can be directed to the corresponding author.

## Supplementary Materials

The following supporting information can be downloaded at: https://www.mdpi.com/article/10.3390/hematolrep17010009/s1, Table S1: General Information; Table S2: Participants’ knowledge about Premarital Screening test; Table S3: Participants’ attitude about Premarital Screening test; Table S4: Association between participants’ knowledge of PMS test and gender; Table S5: Association between participants’ knowledge of PMS test and age group; Table S6: Association between participants’ knowledge of PMS test and education level; Table S7: Association between participants’ knowledge of PMS test and marital status; Table S8: Association between participants’ attitude regarding PMS test and gender; Table S9: Association between participants’ attitude regarding PMS test and age group; Table S10: Association between participants’ attitude regarding PMS test and marital status.
