# Supplementary material for: Awareness and Attitude of the General Population Towards Inherited Hemoglobinopathies in the Premarital Screening Program in the Northern Region of Saudi Arabia
Source: Hematol Rep. 2025 Feb 5;17(1):9. doi: 10.3390/hematolrep17010009 (PMC11855037; doi:10.3390/hematolrep17010009)
Supplement: Supplementary file 1 [file hematolrep-17-00009-s001.zip › hematolrep-3369699-supplementary.pdf]

Table S1. General Information

Items	Levels	Count	Percentage
Are you married to one of your relatives?	Yes	101	21.1%
	No	377	78.9%
	Total	478	100.0%
If the answer of the above question is yes, then what is the degree of relation?	Cousins	68	67.3%
	Other relation	33	32.7%
	Total	101	100.0%
Was there any relationship between your parents before marriage?	Yes	213	44.6%
	No	265	55.4%
	Total	478	100.0%
If the answer of the above question is yes, then what is the degree of relation?	Cousins	150	70.4%
	Other relation	63	29.6%
	Total	213	100.0%
Have you been diagnosed with any of the following inherited diseases (affected with symptoms?)	Sickle cell anemia	3	0.6%
	Thalassemia	2	0.4%
	I have been diagnosed with another genetic disorder that is not listed here	21	4.4%
	I don't have	452	94.6%
	Total	478	100.0%
Have you been diagnosed to be a carrier of any of the following inherited diseases (Carrier with no symptoms?)	Sickle cell anemia	3	0.6%
	Thalassemia	2	0.4%
	I have been diagnosed with another genetic disorder that is not listed here	10	2.1%
	I don't have	463	96.9%
	Total	478	100.0%
Does anyone in your family suffer from any genetic disease?	Yes	49	10.3%
	No	429	89.7%
	Total	478	100.0%
	Sickle cell anemia	4	8.2%
	Thalassemia	4	8.2%

If the answer of the above question is yes, then please identify the disease	He/she has another genetic disorder that is not listed here	41	83.7%
	Total	49	100.0%
If the spouses are carriers of a hereditary blood disease such as thalassemia or sickle cell anemia, in your opinion, what are the possible reasons that prompt the spouses to complete the marriage, knowing the possibility of the risk of their children inheriting the disease	Family or cultural reasons (the necessity to marry within the same family or tribe)	185	38.7%
	Believing in a concept that occurrence of illness is destiny	92	19.2%
	The premarital screening program is complementary and not necessary	35	7.3%
	Other reasons that are not listed here	166	34.7%
	Total	478	100.0%
Have you heard about premarital screening test?	Yes	469	98.1%
	No	9	1.9%
	Total	478	100%
Do you feel that you need more information about premarital screening program so that you can answer questions related to the premarital screening in a clear way?	Yes	379	79.3%
	No	99	20.7%
	Total	478	100.0%

Table S2. Participants' knowledge about Premarital Screening test

S.No	Knowledge items	Response	Count	Percentage
**1**	The premarital screening guarantees having children free from inherited blood disorders	Agree	240	50.2%
		Disagree	112	23.4%
		Undecided	126	26.4%
		Total	478	100.0%
**2**	The premarital screening test screens only some of the common inherited blood disorders (sickle cell anemia and thalassemia)	Agree	193	40.4%
		Disagree	95	19.9%
		Undecided	190	39.7%
		Total	478	100.0%
**3**	Marriage to a relative (Consanguinity) can lead to an increased risk of inherited blood disease in children	Agree	379	79.3%
		Disagree	18	3.8%
		Undecided	81	16.9%
		Total	478	100.0%
**4**	When both of the parents are carriers for the same genetic blood disease, there will be a risk for having an affected child with this disorder	Agree	334	69.9%
		Disagree	28	5.9%
		Undecided	116	24.3%
		Total	478	100.0%
**5**	Marriage could be compatible even if one of the parents is a carrier of the genetic disease	Agree	144	30.1%
		Disagree	168	35.1%
		Undecided	166	34.7%
		Total	478	100.0%
**6**	Do you know that there is a procedure called preimplantation genetic diagnosis that is considered when both spouses are carriers of a genetic disease?	Yes	270	56.5%
		No	208	43.5%
		Total	478	100.0%

Table S3. Participants' attitude about Premarital Screening test

S.No	Attitude items	Response	Count	Percentage
1	Premarital screening should be mandatory before completion of a marriage	Agree	471	98.5%
		Disagree	7	1.5%
		Total	478	100.0%
2	If the couples are at a high risk of having children with genetic blood disorder, their marriage should be prevented.	Agree	364	76.2%
		Disagree	114	23.8%
		Total	478	100.0%
3	If you are in a situation where the person you want to marry is a carrier of one of the genetic blood diseases such as thalassemia or sickle cell anemia while you are healthy, you will proceed to complete the marriage	Agree	110	23.0%
		Disagree	368	77.0%
		Total	478	100.0%
4	If you were in a situation where both of you are carriers of one of the genetic blood diseases, you will proceed to complete the marriage	Agree	82	17.2%
		Disagree	396	82.8%
		Total	478	100.0%
5	You wish to add a test/s that screen/s for all the possible inherited blood disorders so that the premarital screening program becomes wider	Agree	448	93.7%
		Disagree	30	6.3%
		Total	478	100.0%

Table S4. Association between participants' knowledge of PMS test and gender.

S.No	Knowledge items	Response	Gender						Chi Square (P-value)
			Male		Female		Total		
			N	%	N	%	N	%	
1	The premarital screening guarantees having children free from inherited blood disorders	Agree	25	34.2	215	53.1	240	50.2	9.555 (0.008)
		Disagree	20	27.4	92	22.7	112	23.4	
		Undecided	28	38.4	98	24.2	126	26.4	
		Total	73	100.0	405	100.0	478	100.0	
2	The premarital screening test screens only some of the common inherited blood disorders (sickle cell anemia and thalassemia)	Agree	29	39.7	164	40.5	193	40.4	1.656 (0.437)
		Disagree	11	15.1	84	20.7	95	19.9	
		Undecided	33	45.2	157	38.8	190	39.7	
		Total	73	100.0	405	100.0	478	100.0	
3	Marriage to a relative (Consanguinity) can lead to an increased risk of inherited blood disease in children	Agree	47	64.4	332	82.0	379	79.3	12.506 (0.002)
		Disagree	6	8.2	12	3.0	18	3.8	
		Undecided	20	27.4	61	15.1	81	16.9	
		Total	73	100.0	405	100.0	478	100.0	
4	When both of the parents are carriers for the same genetic blood disease, there will be a risk for having an affected child with this disorder	Agree	40	54.8	294	72.6	334	69.9	9.424 (0.009)
		Disagree	7	9.6	21	5.2	28	5.9	
		Undecided	26	35.6	90	22.2	116	24.3	
		Total	73	100.0	405	100.0	478	100.0	
5	Marriage could be compatible even if one of the parents is a carrier of the genetic disease	Agree	31	42.5	113	27.9	144	30.1	9.708 (0.008)
		Disagree	15	20.5	153	37.8	168	35.1	
		Undecided	27	37.0	139	34.3	166	34.7	
		Total	73	100.0	405	100.0	478	100.0	
6	Do you know that there is a procedure called preimplantation genetic diagnosis that is considered when both spouses are carriers of a genetic disease?	Yes	32	43.8	238	58.8	270	56.5	5.609 (0.018)
		No	41	56.2	167	41.2	208	43.5	
		Total	73	100.0	405	100.0	478	100.0	

Table S5. Association between participants' knowledge of PMS test and age group.

S.No	Knowledge items	Response	Age 18-29 N	18-29 %	30-40 N	30-40 %	Over 40 N	Over 40 %	Total N	Total %	Chi Square (P-value)
1	The premarital screening guarantees having children free from inherited blood disorders	Agree	129	45.6	45	63.4	66	53.2	240	50.2	19.034 (0.001)
		Disagree	81	28.6	15	21.1	16	12.9	112	23.4	
		Undecided	73	25.8	11	15.5	42	33.9	126	26.4	
		Total	283	100.0	71	100.0	124	100.0	478	100.0	
2	The premarital screening test screens only some of the common inherited blood disorders (sickle cell anemia and thalassemia)	Agree	104	36.7	37	52.1	52	41.9	193	40.4	14.669 (0.005)
		Disagree	71	25.1	9	12.7	15	12.1	95	19.9	
		Undecided	108	38.2	25	35.2	57	46.0	190	39.7	
		Total	283	100.0	71	100.0	124	100.0	478	100.0	
3	Marriage to a relative (Consanguinity) can lead to an increased risk of inherited blood disease in children	Agree	230	81.3	53	74.6	96	77.4	379	79.3	3.692 (0.449)
		Disagree	12	4.2	3	4.2	3	2.4	18	3.8	
		Undecided	41	14.5	15	21.1	25	20.2	81	16.9	
		Total	283	100.0	71	100.0	124	100.0	478	100.0	
4	When both of the parents are carriers for the same genetic blood disease, there will be a risk for having an affected child with this disorder	Agree	202	71.4	49	69.0	83	66.9	334	69.9	4.910 (0.297)
		Disagree	20	7.1	4	5.6	4	3.2	28	5.9	
		Undecided	61	21.6	18	25.4	37	29.8	116	24.3	
		Total	283	100.0	71	100.0	124	100.0	478	100.0	
5	Marriage could be compatible even if one of the parents is a carrier of the genetic disease	Agree	87	30.7	27	38.0	30	24.2	144	30.1	5.687 (0.224)
		Disagree	104	36.7	20	28.2	44	35.5	168	35.1	
		Undecided	92	32.5	24	33.8	50	40.3	166	34.7	
		Total	283	100.0	71	100.0	124	100.0	478	100.0	
6	Do you know that there is a procedure called preimplantation genetic diagnosis that is considered when both spouses are carriers of a genetic disease?	Yes	155	54.8	40	56.3	75	60.5	270	56.5	1.146 (0.564)
		No	128	45.2	31	43.7	49	39.5	208	43.5	
		Total	283	100.0	71	100.0	124	100.0	478	100.0	

Table S6. Association between participants' knowledge of PMS test and education level.

S.No	Knowledge items	Response	Educational level						Chi Square (P-value)
			Less than university		University or higher		Total		
			N	%	N	%	N	%	
1	The premarital screening guarantees having children free from inherited blood disorders	Agree	4	30.8	236	50.8	240	50.2	2.993 (0.224)
		Disagree	3	23.1	109	23.4	112	23.4	
		Undecided	6	46.2	120	25.8	126	26.4	
		Total	13	100.0	465	100.0	478	100.0	
2	The premarital screening test screens only some of the common inherited blood disorders (sickle cell anemia and thalassemia)	Agree	5	38.5	188	40.4	193	40.4	1.678 (0.432)
		Disagree	1	7.7	94	20.2	95	19.9	
		Undecided	7	53.8	183	39.4	190	39.7	
		Total	13	100.0	465	100.0	478	100.0	
3	Marriage to a relative (Consanguinity) can lead to an increased risk of inherited blood disease in children	Agree	6	46.2	373	80.2	379	79.3	9.125 (0.010)
		Disagree	1	7.7	17	3.7	18	3.8	
		Undecided	6	46.2	75	16.1	81	16.9	
		Total	13	100.0	465	100.0	478	100.0	
4	When both of the parents are carriers for the same genetic blood disease, there will be a risk for having an affected child with this disorder	Agree	4	30.8	330	71.0	334	69.9	9.813 (0.007)
		Disagree	2	15.4	26	5.6	28	5.9	
		Undecided	7	53.8	109	23.4	116	24.3	
		Total	13	100.0	465	100.0	478	100.0	
5	Marriage could be compatible even if one of the parents is a carrier of the genetic disease	Agree	4	30.8	140	30.1	144	30.1	1.058 (0.589)
		Disagree	3	23.1	165	35.5	168	35.1	
		Undecided	6	46.2	160	34.4	166	34.7	
		Total	13	100.0	465	100.0	478	100.0	
6	Do you know that there is a procedure called preimplantation genetic diagnosis that is considered when both spouses are carriers of a genetic disease?	Yes	4	30.8	266	57.2	270	56.5	3.595 (0.058)
		No	9	69.2	199	42.8	208	43.5	
		Total	13	100.0	465	100.0	478	100.0	

Table S7. Association between participants' knowledge of PMS test and marital status.

S.No	Knowledge items	Response	Marital status						P-value
			Never married		Married/ married before		Total		
			N	%	N	%	N	%	
1	The premarital screening guarantees having children free from inherited blood disorders	Agree	125	47.9	115	53.0	240	50.2	2.323 (0.313)
		Disagree	68	26.1	44	20.3	112	23.4	
		Undecided	68	26.1	58	26.7	126	26.4	
		Total	261	100.0	217	100.0	478	100.0	
2	The premarital screening test screens only some of the common inherited blood disorders (sickle cell anemia and thalassemia)	Agree	91	34.9	102	47.0	193	40.4	16.941 (<0.001)
		Disagree	69	26.4	26	12.0	95	19.9	
		Undecided	101	38.7	89	41.0	190	39.7	
		Total	261	100.0	217	100.0	478	100.0	
3	Marriage to a relative (Consanguinity) can lead to an increased risk of inherited blood disease in children	Agree	216	82.8	163	75.1	379	79.3	4.399 (0.111)
		Disagree	9	3.4	9	4.1	18	3.8	
		Undecided	36	13.8	45	20.7	81	16.9	
		Total	261	100.0	217	100.0	478	100.0	
4	When both of the parents are carriers for the same genetic blood disease, there will be a risk for having an affected child with this disorder	Agree	188	72.0	146	67.3	334	69.9	5.400 (0.067)
		Disagree	19	7.3	9	4.1	28	5.9	
		Undecided	54	20.7	62	28.6	116	24.3	
		Total	261	100.0	217	100.0	478	100.0	
5	Marriage could be compatible even if one of the parents is a carrier of the genetic disease	Agree	78	29.9	66	30.4	144	30.1	1.201 (0.549)
		Disagree	97	37.2	71	32.7	168	35.1	
		Undecided	86	33.0	80	36.9	166	34.7	
		Total	261	100.0	217	100.0	478	100.0	
6	Do you know that there is a procedure called preimplantation genetic diagnosis that is considered when both spouses are carriers of a genetic disease?	Yes	142	54.4	128	59.0	270	56.5	1.011 (0.315)
		No	119	45.6	89	41.0	208	43.5	
		Total	261	100.0	217	100.0	478	100.0	

Table S8. Association between participants' attitude regarding PMS test and gender.

S.No	Attitude items	Response	Gender						P-value
			Male		Female		Total		
			N	%	N	%	N	%	
**1**	Premarital screening should be mandatory before completion of a marriage	Agree	71	97.3	400	98.8	471	98.5	0.971 (0.324)
		Disagree	2	2.7	5	1.2	7	1.5	
		Total	73	100.0	405	100.0	478	100.0	
**2**	If the couples were at a high risk of having children with genetic blood disorder, their marriage should be prevented	Agree	53	72.6	311	76.8	364	76.2	0.597 (0.440)
		Disagree	20	27.4	94	23.2	114	23.8	
		Total	73	100.0	405	100.0	478	100.0	
**3**	If you are in a situation where the person you want to marry is a carrier of one of the genetic blood diseases such as thalassemia or sickle cell anemia while you are healthy, you will proceed to complete the marriage	Agree	24	32.9	86	21.2	110	23.0	4.732 (0.030)
		Disagree	49	67.1	319	78.8	368	77.0	
		Total	73	100.0	405	100.0	478	100.0	
**4**	If you were in a situation where both of you are carriers of one of the genetic blood diseases, you will proceed to complete the marriage	Agree	22	30.1	60	14.8	82	17.2	10.217 (0.001)
		Disagree	51	69.9	345	85.2	396	82.8	
		Total	73	100.0	405	100.0	478	100.0	
**5**	You wish to add a test/s that screen/s for all the possible inherited blood disorders so that the premarital screening program becomes wider	Agree	69	94.5	379	93.6	448	93.7	0.093 (0.760)
		Disagree	4	5.5	26	6.4	30	6.3	
		Total	73	100.0	405	100.0	478	100.0	

Table S9. Association between participants' attitude regarding PMS test and age group.

S.No	Attitude items	Response	Age 18-29 N	18-29 %	30-40 N	30-40 %	Over 40 N	Over 40 %	Total N	Total %	Chi Square (P-value)
1	Premarital screening should be mandatory before completion of a marriage	Agree	278	98.2	69	97.2	124	100.0	471	98.5	2.922 (0.23)2
		Disagree	5	1.8	2	2.8	0	0.0	7	1.5	
		Total	283	100.0	71	100.0	124	100.0	478	100.0	
2	If the couples were at a high risk of having children with genetic blood disorder, their marriage should be prevented	Agree	203	71.	58	81.7	103	83.1	364	76.2	7.507 (0.023)
		Disagree	80	28.3	13	18.3	21	16.9	114	23.8	
		Total	283	100.0	71	100.0	124	100.0	478	100.0	
3	If you are in a situation where the person you want to marry is a carrier of one of the genetic blood diseases such as thalassemia or sickle cell anemia while you are healthy, you will proceed to complete the marriage	Agree	73	25.8	20	28.2	17	13.7	110	23.0	8.359 (0.015)
		Disagree	210	74.2	51	71.8	107	86.3	368	77.0	
		Total	283	100.0	71	100.0	124	100.0	478	100.0	
4	If you were in a situation where both of you are carriers of one of the genetic blood diseases, you will proceed to complete the marriage	Agree	64	22.6	9	12.7	9	7.3	82	17.2	15.484 (<0.001)
		Disagree	219	77.4	62	87.3	115	92.7	396	82.8	
		Total	283	100.0	71	100.0	124	100.0	478	100.0	
5	You wish to add a test/s that screen/s for all the possible inherited blood disorders so that the premarital screening program becomes wider	Agree	260	91.9	69	97.2	119	96.0	448	93.7	4.154 (0.125)
		Disagree	23	8.1	2	2.8	5	4.0	30	6.3	
		Total	283	100.0	71	100.0	124	100.0	478	100.0	

Table S10. Association between participants' attitude regarding PMS test and marital status.

S.No	Attitude items	Response	Marital status						Chi Square (P-value)
			Never married		Married/ married before		Total		
			N	%	N	%	N	%	
1	Premarital screening should be mandatory before completion of a marriage	Agree	256	98.1	215	99.1	471	98.5	0.811 (0.368)
		Disagree	5	1.9	2	0.9	7	1.5	
		Total	261	100.0	217	100.0	478	100.0	
2	If the couples were at a high risk of having children with genetic blood disorder, their marriage should be prevented	Agree	189	72.4	175	80.6	364	76.2	4.420 (0.036)
		Disagree	72	27.6	42	19.4	114	23.8	
		Total	261	100.0	217	100.0	478	100.0	
3	If you are in a situation where the person you want to marry is a carrier of one of the genetic blood diseases such as thalassemia or sickle cell anemia while you are healthy, you will proceed to complete the marriage	Agree	69	26.4	41	18.9	110	23.0	3.805 (0.051)
		Disagree	192	73.6	176	81.1	368	77.0	
		Total	261	100.0	217	100.0	478	100.0	
4	If you were in a situation where both of you are carriers of one of the genetic blood diseases, you will proceed to complete the marriage	Agree	56	21.5	26	12.0	82	17.2	7.484 (0.006)
		Disagree	205	78.5	191	88.0	396	82.8	
		Total	261	100.0	217	100.0	478	100.0	
5	You wish to add a test/s that screen/s for all the possible inherited blood disorders so that the premarital screening program becomes wider	Agree	238	91.2	210	96.8	448	93.7	6.286 (0.012)
		Disagree	23	8.8	7	3.2	30	6.3	
		Total	261	100.0	217	100.0	478	100.0	
